# A Pathophysiological Insight into Sepsis and Its Correlation with Postmortem Diagnosis

**DOI:** 10.1155/2016/4062829

**Published:** 2016-04-27

**Authors:** C. Pomara, I. Riezzo, S. Bello, D. De Carlo, M. Neri, E. Turillazzi

**Affiliations:** Department of Clinical and Experimental Medicine, Section of Forensic Pathology, Ospedale Colonnello D'Avanzo, University of Foggia, Viale degli Aviatori 1, 71100 Foggia, Italy

## Abstract

*Background*. Sepsis is among the leading causes of death worldwide and is the focus of a great deal of attention from policymakers and caregivers. However, sepsis poses significant challenges from a clinical point of view regarding its early detection and the best organization of sepsis care. Furthermore, we do not yet have reliable tools for measuring the incidence of sepsis. Methods based on analyses of insurance claims are unreliable, and postmortem diagnosis is still challenging since autopsy findings are often nonspecific.* Aim*. The objective of this review is to assess the state of our knowledge of the molecular and biohumoral mechanisms of sepsis and to correlate them with our postmortem diagnosis ability.* Conclusion*. The diagnosis of sepsis-related deaths is an illustrative example of the reciprocal value of autopsy both for clinicians and for pathologists. A complete methodological approach, integrating clinical data by means of autopsy and histological and laboratory findings aiming to identify and demonstrate the host response to infectious insults, is mandatory to illuminate the exact cause of death. This would help clinicians to compare pre- and postmortem findings and to reliably measure the incidence of sepsis.

## 1. Introduction

Sepsis can be defined as a syndrome of dysregulated inflammation caused by the failure of infection control and containment mechanisms. It should be considered a major public health problem since it affects millions of people worldwide each year, with an incidence which is dramatically increasing [1]. It accounts for most deaths in critically ill patients [2–4]. The hospital mortality of patients with sepsis ranges from 28.3 to 41.1% in North America and Europe [5, 6].

Consequently, considerable attention is dedicated to sepsis by policymakers and caregivers. However, these efforts are tempered by several limitations. First of all, difficulty in defining sepsis still exists due to the emerging biological insights and reported variation in epidemiology. Recently the Third International Consensus Definitions Task Force defined sepsis as “life-threatening organ dysfunction due to a dysregulated host response to infection” [7, 8]; however the performance of clinical criteria for this sepsis definition is unknown [7]. Furthermore, sepsis poses heavy challenges from a clinical point of view regarding its early detection and the best organization of sepsis care [6, 9]. Finally, we do not yet have reliable tools for measuring the incidence of sepsis. Methods based on analyses of insurance claim data using sepsis-specific codes or separate codes for infection and organ dysfunction are unreliable in informing or measuring the effects of policy changes [1], and the postmortem diagnosis of sepsis is still challenging as the results of postmortem investigations often show a relative paucity of significant macroscopic and histopathological findings [10, 11]. Apart from the possibility of demonstrating infected sites in the internal organs (i.e., septicopyaemic abscess), the inflammatory organ changes observed at autopsy are mediated by the endogenous inflammatory mediators that are neither specific nor sensitive with regard to sepsis [11]. These can also be demonstrated in different clinical conditions going along with systemic inflammatory response syndrome (SIRS) or prolonged ischemia [11]. Furthermore, the presence of a pathogen in the blood or tissues does not necessarily indicate that the complex syndrome of sepsis has occurred [12]. These issues are further demonstrated by the discrepancies existing between clinical and postmortem diagnosis of sepsis [13–15]. Despite the fact that the latter is still a diagnosis of exclusion, sepsis is an intriguing field of interest in autopsy practice, and the diagnosis of sepsis-related deaths is an illustrative example of the reciprocal value of autopsy both for clinicians and for pathologists. Upon studying these deaths, the pathologist needs to approach the complex pathophysiological mechanisms underlying sepsis. This may lead in turn to an increased understanding of the pathogenic hypotheses and to the accuracy of existing diagnostic tools to be checked [16]. Autopsies should still serve as a very important part of quality control in clinical diagnosis and treatment of sepsis [17, 18]. Last but not least, the issue of the diagnostic reliability of fatal sepsis is even more pivotal. One important area which has seen a rising number of clinical negligence claims comprises healthcare-associated infections and sepsis [19].

The objective of this review is to assess the state of our knowledge of the molecular and biohumoral mechanisms of sepsis and to correlate them with our postmortem diagnosis ability.

## 2. The Inflammatory Response

The septic response is an extremely complex chain of events involving inflammatory and anti-inflammatory processes, humoral and cellular reactions, and circulatory changes. When an infectious insult occurs, it initiates a series of events, resulting in the release of inflammation mediators which involves both a local reaction and a systemic response and which, finally, could impact on organs function [20].

Sepsis triggers the production of a diverse array of cytokines that are proinflammatory and anti-inflammatory [21, 22]. Cytokines are low molecular weight compounds which are considered potent positive and negative regulators of inflammation. Some cytokines possess proinflammatory effects such as tumour necrosis factor- (TNF-) alpha, interleukin- (IL-) 1, and interleukin-8, while others have anti-inflammatory effects including IL-10 and IL-1 receptor antagonist, and some are supposed to act as pro- and anti-inflammatory IL-6 as an example [23].

Proinflammatory cytokines trigger a beneficial response, such as increased local coagulation, limited tissue damage, and elimination of the pathogen. Overwhelming production of these proinflammatory cytokines, however, can be very dangerous in that excessive cytokines destroy the normal regulation of the immune response and induce pathological inflammatory disorders, such as capillary leakage, tissue injury, and organ failure. Similarly, the anti-inflammatory cytokines play a critical role in regulating overall immune response, in establishing homeostasis, and in checking the effects of the proinflammatory ones in order to solve inflammation and heal tissue. Therefore, their dysregulation can also trigger pathogenesis [24], since it has been shown that an intense anti-inflammatory response may induce a state of immunosuppression in patients with sepsis [25]. When a state of anti-inflammatory predominance occurs, monocytes are deactivated. This results in reduced antigen presentation and decreased production of proinflammatory cytokines, leading to pathogen persistence and further fuelling the inflammatory process [21, 22, 25]. A tightly regulated balance in the cytokine network, which comprises pro- and anti-inflammatory cytokines, is crucial for eliminating invading pathogens on the one hand and restricting excessive, tissue-damaging inflammation on the other [26].

In brief, when an infectious insult occurs, pattern recognition receptors (PRRs), which are expressed on epithelial barriers as well as on resident immune cells such as dendritic cells and macrophages, detect invading microorganisms. A specific family of PRRs named toll-like receptors (TLRs) recognizes conserved macromolecular motifs from microorganisms, called pathogen-associated molecular patterns (PAMPs). The stimulation of TLRs or the nucleotide oligomerization domain NOD-like receptor (NLR) family of intracellular PRRs results in the triggering of downstream signalling cascades. Depending on the particular receptor engaged, this process leads to the activation of a transcriptional response programme that includes nuclear factor *κ*B (NF-*κ*B), followed by the production and secretion of cytokines, chemokines, and nitric oxide [21, 22]. Cytokines usually bind their specific receptors, induce signalling pathways, and thus regulate immune responses and other cell functions [24].

Therefore, different types of cells, tissues/organs, or protein/other molecules may function as effectors, modulating the immune response through various pro- or anti-inflammatory mediators. Resident macrophages and polymorphonuclear cells (PMCs) initiate the primary host response to the invading microorganisms. They are responsible for the primary phagocytosis and subsequent activation and recruitment of granulocytes and monocytes. Leukostasis of neutrophils (the so-called leucocyte sticking) in the liver sinusoids, in the pulmonary vessels, and so forth is the common histological counterpart of this phenomenon [11]. Proliferation of astrocytes and microglial cells is a common postmortem histological finding in septic patients. However, it is highly nonspecific as it may reflect several kinds of insults, including ischemia [11].

Endothelial cells (ECs) activation occurs during sepsis. The interaction between ECs and leukocytes, a hallmark of the inflammatory process, comprises adhesive and migratory molecular events including low-affinity transient and reversible rolling adhesions, integrin-dependent firm adhesive interactions, and migratory events of the leukocytes through the endothelium and, finally, in the interstitial space [27, 28]. A variety of chemical mediators secreted from inflamed tissue and the entire process of leukocyte-endothelial cell adhesion are regulated by the sequential activation of different families of adhesion molecules that are expressed on the surface of leukocytes and ECs. Lectin-like adhesion glycoproteins, selectins, mediate leukocyte rolling. The firm adhesion and subsequent transendothelial migration of leukocytes are mediated by the interaction of integrins expressed by leukocytes with immunoglobulin-like adhesion molecules on ECs, for example, intercellular cell adhesion molecules (ICAM) 1–5, vascular cell adhesion molecule-1 (VCAM-1), and the junctional adhesion molecules (JAMs), which are expressed on endothelial and other cells [29, 30]. Recently, EC-active molecules (i.e., the angiopoietin pathway, Ang-2, soluble fms-like tyrosine kinase-1) have been proposed to correlate significantly with organ dysfunction and mortality in patients with sepsis [31, 32].

Sepsis is also associated with robust activation of the complement system, as demonstrated by the presence of complement activation products (C3a, C5a, and C5b-9) in the plasma [33]. C3a and C5a anaphylatoxins are small cleavage products of C3 and C5 and possess proinflammatory activities. C5a especially reacts with its receptors on phagocytes (neutrophils, macrophages) and on a variety of organs to trigger numerous biological effects. These include increasing vascular permeability and inducing smooth muscle contraction, inducing chemotaxis of PMCs, monocytes, and other cell types [34, 35]. In experimental studies on animal models (rats or mice with cecal ligation and puncture, CLP), both rabbit polyclonal neutralizing antibodies and mouse monoclonal antibodies that neutralized C5a were highly effective in attenuating the parameters of sepsis (clinical symptoms, evidence of multiorgan failure, MOF, consumptive coagulopathy, innate immune functions, apoptosis, etc.), resulting in greatly improved survival [36, 37]. The effects of C5a contribute to immunoparalysis, MOF, the apoptosis of thymocytes and adrenal medullary cells, and imbalances in the coagulation system [38]. In addition, C5a is involved in the development of septic cardiomyopathy and severe left ventricular dysfunction [39]. At autopsy, the left ventricle is often dilated and the ventricular walls have a flaccid appearance [11].

Other products derived from activation of the complement system play an important role in sepsis, such as C3b (from C3), which is a key opsonisation factor that reacts with phagocytes receptors to favour internalization of bacteria and their subsequent elimination. The membrane attack complex (C5b-9) causes lysis of Gram-negative bacteria [38].

**Figure 1:**
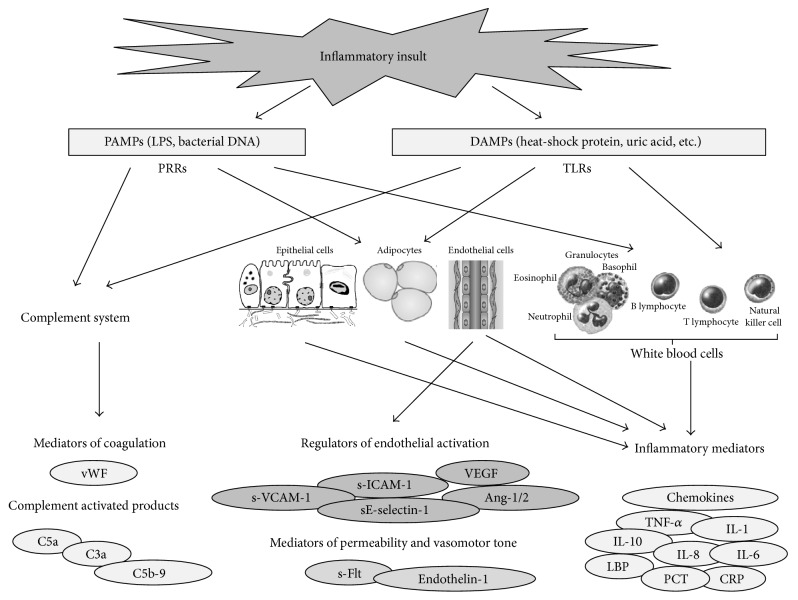
Schematic of events associated with major mediators of cytokine cascade on initiation of sepsis. The release of a large amount of pathogen and damage associated molecular patterns (PAMPs and DAMPs) from invading microorganisms and/or damaged host tissue, respectively, results in the overstimulation of pattern-recognition receptors (PRRs) of immune cells. This process activates an inflammatory cascade in which large amounts of cytokines are released into the body. Macrophages and endothelial cells are then hyperactivated by the unusually large quantity of circulating cytokines. The activation of macrophages and endothelial cells results in the release of more cytokines, exacerbating the inflammatory response. The profound proinflammatory response is counteracted by certain anti-inflammatory cytokines, including IL-10, transforming growth factor-*β* (TGF-*β*), and IL-4, which attempt to restore immunological equilibrium.

Finally, sepsis also affects other biological systems, such as the coagulation system and the autonomic nervous system [39]. In the clinical setting of sepsis, dysregulation of the coagulation cascade results in major complications. The extent of activation of the coagulation cascade during sepsis can range from an insignificant level to the occurrence of disseminated intravascular coagulation (DIC). When DIC occurs, at autopsy various degree of haemorrhage can be observed on the skin, on mucocutaneous surfaces and serous membranes, and, finally, in parenchymal organs [10, 11]. Increasing evidence points to an extensive cross talk between inflammation and coagulation, in which the protease activated cell receptors play an important role (Figure 1).

## 3. The Concept of “Compartmentalization” of the Inflammatory Response in Sepsis

A fundamental step in the comprehension of sepsis pathophysiology is to appreciate that the inflammatory response varies from one compartment to another, and not all compartments behave similarly [40]. Chinnaiyan et al. supported this concept by studying gene expression in different tissues in CLP model of sepsis in rats. They showed that the sepsis response elicited gene expression profiles that were either organ specific, common to more than one organ, or distinctly opposite in some organs [41].

Each organ has a distinctive molecular response to systemic inflammation. Several experimental pieces of data confirm this observation. For example, neutrophil sequestration in lung and liver resulted differently regulated by chemokines in a murine experimental model of peritonitis [42]. After injection of LPS (lipopolysaccharide) in a mice model, NF-*κ*B activation in liver was mediated through TNF and IL-1 receptor-dependent pathways, but, in the lung, LPS induced NF-*κ*B activation was largely independent of these receptors [43]. Although data are still missing in humans, mouse alveolar macrophages do not produce IL-10 [44], do not express TLR9, and are thus insensitive to bacterial DNA [45] and fail to produce IFN-*β* in response to TLR4 and TLR3 agonists [46], thus demonstrating that, in this animal model, alveolar macrophages behave differently to other types of macrophages.

Through the bloodstream a strict cross talk exists between the different organs and compartments. Both pro- and anti-inflammatory mediators are present concomitantly in the bloodstream, evoking different responses in the various organs. The latter, in turn, respond through the local production of different mediators and the activation of different cellular types [40]. Most tissues contribute to the release of inflammatory mediators and there is local activation of intracellular signalling pathways [40].

**Figure 2:**
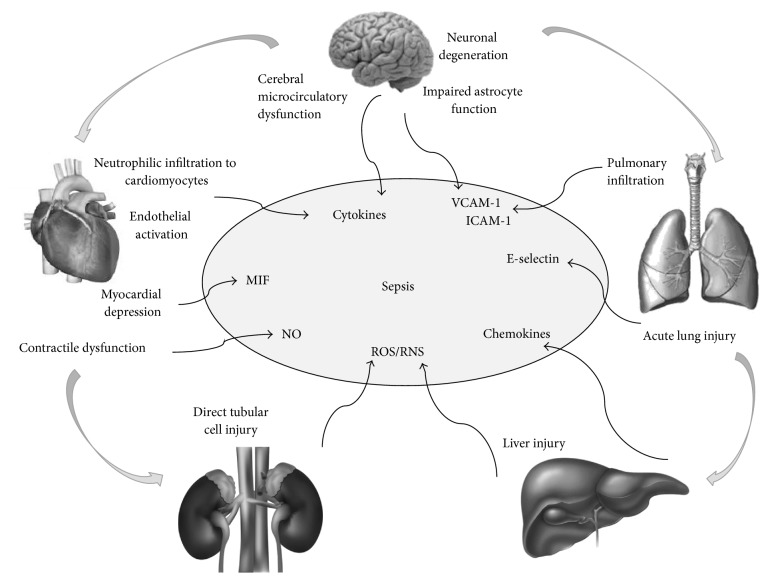
A schematic representing the involvement of different organs in sepsis. During sepsis large amounts of inflammatory mediators are found within the bloodstream. They can act on different organs and induce tissue injury that in turn will favour further production of inflammatory mediators. Cross talk between the different organs and tissues is further mediated by the local delivery of mediators that can amplify or limit the inflammatory response (MIF, macrophage migration inhibitory factor; NO, nitric oxide; ROS, reactive oxygen species; RNS, reactive nitrogen species; VCAM-1, vascular adhesion molecule-1; and ICAM-1, intercellular adhesion molecule-1).

This concept is of paramount clinical importance as the various organs may respond differently to therapeutic strategies. On the other hand, upon approaching sepsis-related deaths, pathologists must keep in mind that tissue injury can be initiated remotely from an insult in a faraway site and that all organs and compartments may be involved (Figure 2).

Different organs and systems are interconnected via humoral and biochemical interactions and are clustered into functional modules sharing many common pathophysiological mechanisms. Diagnostic postmortem strategies based on the measurement of compartmentalized mediators may prove useful as a diagnostic strategy [47, 48].

## 4. Microcirculation and Microvesicles

Sepsis is a disease of microcirculation [49]. Nuclear vacuolization, cytoplasmic swelling and protrusion, cytoplasmic fragmentation, and various degrees of endothelial detachment from its basement membrane have been demonstrated during sepsis [50, 51]. Endothelial physical disruption leads to an extravascular leak of protein-rich oedema and polymorphonuclear cells (PMNs) influx into organs. Furthermore, endothelial damage may induce leukocyte and platelet aggregation, as well as aggravation of coagulopathy, thus favouring impaired perfusion, tissue hypoxia, and subsequent organ dysfunction [51, 52].

Deleterious effects on the vascular function are mediated by increased synthesis of inflammatory cytokines and chemokines and increased expression of endothelial adhesion molecules [49–51]. Microvascular endothelial cells (MVECs) that are critical modulators of blood flow and microvascular function are principal targets of the systemic inflammation of sepsis [52].

Microvasculature dysfunction in sepsis is almost ubiquitous. Pulmonary MVECs injury and barrier dysfunction result in the leak of protein-rich fluid and circulating neutrophils into the pulmonary interstitium and alveolar spaces [52–54]. Recently it has been demonstrated that septic pulmonary microvascular barrier dysfunction is associated with significant pulmonary MVECs death, which is largely apoptotic [52]. Experimental observations provide proof that septic acute kidney injury (AKI) can occur in the setting of renal hyperaemia and that ischemia is not necessarily present. Nonhemodynamic mechanisms of cell injury are likely to be at work, due to a combination of immunologic, toxic, and inflammatory factors that may affect the microvasculature and the tubular cells [55–58]. There is evidence that adhesion molecule activation, both on the renal endothelium and on epithelial cells, leads to enhanced leukocyte adhesion, followed by the influx of activated leukocytes into the renal interstitium [57]. Finally, renal mitochondrial dysfunction has been demonstrated in CLP murine model of sepsis leading to a decrease in renal* complexes I* and* II/III* respiration, MnSOD (manganese superoxide dismutase) activity, and ATP levels. This was associated with increased mitochondrial superoxide levels, impaired renal microcirculation, and impaired renal function [59]. Oxidant generation by the renal tubules and renal microvascular failure are early events, which lead to AKI [60–62]. Cerebral microcirculatory dysfunction has been demonstrated in various experimental models of sepsis [63], and it is thought to be one of the main pathophysiological mechanisms leading to brain damage also in humans [64]. Both endotoxin, or more accurately termed bacterial lipopolysaccharide (LPS), and proinflammatory cytokines induce the expression of CD40, VCAM-1 or ICAM-1, and E-selectin on human brain microvessel endothelial cells [65–69].

Finally, in recent years a growing body of evidence has been established regarding the role of microvesicles (MVs) in sepsis [70]. These extracellular vesicles are released in the extracellular environment through a membrane reorganization and blebbing process following cell activation or apoptosis. They constitute a storage pool of bioactive effectors with varied cellular origins and are able to act as intercellular messengers or effectors through multiple amplification and regulatory loops affecting vascular cells functions [71]. Thus, MVs contribute to the spread of inflammatory and prothrombotic vascular status. They may also affect smooth muscle tissue through adhesion molecules, activation of NF-*κ*B, and the expression of inducible nitric oxide synthase and cyclooxygenase-2, with an increase in nitric oxide and vasodilator prostanoids, leading to arterial hyporeactivity [72–74].

**Figure 3:**
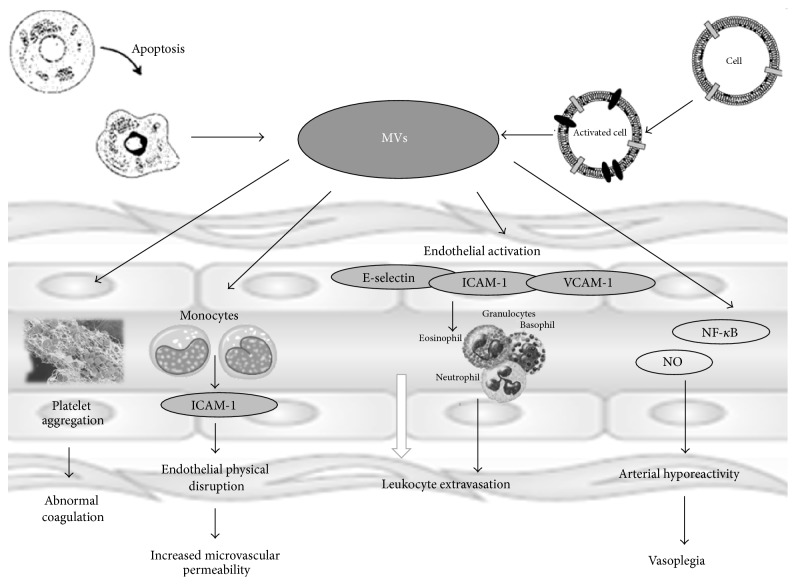
A schematic representing microvesicles (MVs) functions. MVs are released in the extracellular environment through a membrane reorganization and blebbing process following cell activation or apoptosis. They contribute to the spread of inflammatory and prothrombotic vascular status and they may affect smooth muscle tissue through adhesion molecules (E-selectin, ICAM, and VCAM) activation of nuclear factor *κ*B and the expression of inducible nitric oxide synthase and cyclooxygenase-2, with an increase in nitric oxide (NO) and vasodilator prostanoids, leading to arterial hyporeactivity.

In recent years, the analysis of circulating-cell-derived MVs has become more defined and clinically more useful, and several groups suggest that it may enter mainstream clinical testing [75, 76]. Endothelial-derived microvesicles are considered relevant biomarkers of septic shock-induced DIC and have been proposed as a significant diagnostic tool to evaluate early vascular injury [74]. An increase of platelet derived microvesicles has been demonstrated in the development of sepsis-related renal impairment [77] and in severe fungal sepsis [78]. Finally, MVs released from peripheral blood PMNs have a similar size and orientation but differ in protein composition and functional properties. These affect endothelial cells, platelets, monocytes, and macrophages. They also show antibacterial properties since they are capable of a significant reduction in bacterial growth (Figure 3) [79–81].

## 5. The Blood Compartment

Blood compartment plays a key role in the inflammatory response. Waves of mediators with both anti- and proinflammatory properties are detectable in the plasma of septic patients. Many of these are considered reliable markers for* in vita* diagnosis of sepsis and some have been proposed also as biological markers of the severity of sepsis, but none alone is entirely specific for infection because they can be also detected in the absence of infection [82]. The future direction of research is most likely to focus on the use of panels or combinations of markers with clinical signs. Some biomarkers may also be useful for prognosis and guiding therapy [83–86]. However, nowadays the ideal biomarker, with high sensitivity and specificity and cost effectiveness and with definite cut-off ranges and time of blood sampling, is yet to be found [87].

As molecular and humoral mechanisms are thought to play a key part in the pathophysiology of sepsis, postmortem diagnostic strategies based on the concentration of such mediators have been investigated.

Many authors propose the detection of procalcitonin (PCT) as a useful and reliable marker of sepsis in routine autopsy investigations [88–91]. PCT, a prohormone composed of 116 amino acids, is the precursor of the calcium homoeostasis hormone calcitonin, which is found in thyroid C cells and pulmonary endocrine cells. Clinically relevant levels of PCT influence the immunologic responses that contribute to systemic inflammatory responses and septic shock. Many studies have indicated that PCT is an excellent marker of bacterial infection in patients with sepsis and its related conditions. Bode-Jänisch et al. outline that, at PCT levels <2 ng/mL, bacterial sepsis or septic shock can almost certainly be excluded as cause of death [89]. PCT levels ≥10 ng/mL can be detected occasionally in conditions other than sepsis. A final assessment should therefore take into account the PCT levels, autopsy results, and the histopathological and microbiological findings [91]. Other authors indicate high diagnostic accuracy for both lipopolysaccharide-binding protein (LBP) and PCT, considered individually and combined, in detecting sepsis-related outcomes in postmortem [92].

However, due to the detectability of high levels of PCT also in aseptic inflammation (i.e., chronic inflammatory and autoimmune conditions, myocardial infarction, etc.) [93–103], a pressing need to identify additional biomarkers is evident [91].

As demonstrated by the studies mentioned above, much attention has been focused on cytokines and other mediators as diagnostic tools in many diseases and they certainly hold promise also for the discovery of reliable postmortem biomarkers of sepsis.

Soluble interleukin-2 receptor (sIL-2R) and LBP seem to represent appropriate diagnostic tools for the postmortem diagnosis of sepsis [104]. Postmortem IL-6 and C-reactive protein (CRP) serum levels were investigated by Tsokos et al. in sepsis and nonseptic fatalities and both IL-6 and CRP serum concentrations seem to be suitable biochemical markers of sepsis [105]. However, since pathological conditions other than sepsis (trauma, burn injury, etc.) may be associated with elevated IL-6 and/or CRP levels, the authors themselves warn about the need to rule out such conditions upon interpreting postmortem values of IL-6 and CRP [105].

Other biomarkers of potential relevance to sepsis/septic shock diagnostics have been proposed more recently.

Triggering receptor expressed on myeloid cells-1 (TREM-1) is a recently discovered member of the immunoglobulin superfamily of receptors that is specifically expressed on the surfaces of neutrophils and monocytes and upregulated in bacterial sepsis. This is associated with a marked plasma elevation in the soluble form of this molecule (sTREM-1). Studies have indicated that sTREM-1 could be a valuable diagnostic biomarker for sepsis [106]. Palmiere et al. investigated sTREM-1 concentration in the serum of patients who died from sepsis and found that when individually considered, it did not provide better sensitivity and specificity than PCT in detecting sepsis. However, simultaneous assessment of PCT and sTREM-1 in postmortem serum could improve diagnostic accuracy [87]. Results from septic patients in intensive care units found that sTREM-1, PCT, and CRP levels indicate infection, while sTREM-1 and PCT levels predict prognosis. Moreover, sTREM-1 appears to be the best indicator for the diagnosis of sepsis and assessment of prognosis of blood culture-positive bacteremia [107].

Presepsin (sCD14-ST) is a soluble N-terminal fragment of protein CD14 which is released into circulation during monocyte activation on the recognition of LPS from infectious agents [108]*;* it shows promise for diagnostic and prognostic purposes in septic patients [109]. It has been investigated in sepsis-related death. The results show that even though increases in both PCT and sCD14-ST concentrations were observed in the control cases, coherent PCT and sCD14-ST results in cases with suspected sepsis allowed the diagnosis to be confirmed. Conversely, no relevant correlation was identified between postmortem serum and pericardial fluid sCD14-ST levels in either the septic or control groups [110].

Endocan (endothelial cell-specific molecule-1), a 50-kDa dermatan sulphate proteoglycan, expressed by endothelial cells in lung and kidney, can be detected at low levels in the serum of healthy subjects. Increased concentrations were described in patients with sepsis, severe sepsis, and septic shock compared to healthy individuals, with serum concentrations related to the severity of illness [111]. Palmiere and Augsburger found that postmortem serum endocan concentrations were significantly higher in sepsis fatal cases, with values ranging from 0.519 ng/mL to 6.756 ng/mL, while, in most patients of the control group, endocan was undetectable. The authors argue that endocan could be considered a suitable biological parameter for the detection of sepsis-related deaths in forensic pathology routine [112].

Neopterin (D-erytro-1′,2′,3′-trihydroxypropylterin), a biochemical product of guanosine triphosphate pathway, has been proposed to aid in the diagnosis of bacterial [113] and viral infections [114]. Also in the forensic literature neopterin has been proposed as a marker of inflammatory diseases [115–117]. Postmortem serum neopterin levels over 500 nmol/L were observed in bacterial and viral infection cases as well as in delayed deaths due to trauma [118]. For this reason, the specificity of neopterin as a clinical marker of bacterial sepsis is limited [119].

Conclusively, there is a strong body of evidence that postmortem concentration of serum cytokines and other mediators of inflammation may be an area of great interest with exciting diagnostic possibilities for sepsis/septic shock-related deaths [118, 119]. However, at present, an ideal clinical and postmortem marker of sepsis does not exist [118].

In a previous review, Pierrakos and Vincent identified nearly 180 distinct molecules that have been proposed as potential biological markers of sepsis [83]. However, only 20% of these biomarkers have been assessed specifically in appropriate studies for use in the diagnosis of sepsis [84].

## 6. Conclusion

The difficulties of the postmortem diagnosis of death due to sepsis are well known. The major limitation is the poor specificity of macroscopic and routine histological findings encountered in such cases. Due to the complex molecular and cellular mechanisms underlying sepsis, proof of the presence of germs alone cannot be of evidential value in establishing a causal relationship between infection and outcome. Thus, the old saying by William Osler (1849 to 1919) “except on few occasions, the patient appears to die from the body's response to infection rather than from the infection” is still true [84].

A complete methodological approach, integrating clinical data by means of autopsy and histological and laboratory findings aiming to identify and demonstrate the host response to infectious insult, is mandatory. Such an approach would be likely to produce an accurate objective surveillance of deaths due to sepsis and improve our knowledge of the clinical-pathological correlation in sepsis, thus contributing to the evaluation of the effectiveness of therapies. Finally, autopsy is a critical tool for protection from false liability claims and settling valid claims quickly and fairly.
